# Molecular characterization of circulating tumor cells from patients with metastatic breast cancer reflects evolutionary changes in gene expression under the pressure of systemic therapy

**DOI:** 10.18632/oncotarget.17271

**Published:** 2017-04-20

**Authors:** Kristina E. Aaltonen, Vendula Novosadová, Pär-Ola Bendahl, Cecilia Graffman, Anna-Maria Larsson, Lisa Rydén

**Affiliations:** ^1^ Department of Clinical Sciences Lund, Division of Oncology and Pathology, Lund University, Lund, Sweden; ^2^ Institute of Biotechnology, BIOCEV Centre, Czech Academy of Sciences, Vestec, Czech Republic; ^3^ Skåne Department of Oncology, Skåne University Hospital, Lund, Sweden; ^4^ Department of Translational Cancer Research, Lund University, Lund, Sweden; ^5^ Department of Clinical Sciences Lund, Division of Surgery, Lund University, Lund, Sweden; ^6^ Department of Surgery, Skåne University Hospital, Malmö, Sweden

**Keywords:** metastatic breast cancer, circulating tumor cells, gene expression, human epidermal growth factor receptor 2 (HER2), estrogen receptor (ER)

## Abstract

Resistance to systemic therapy is a major problem in metastatic breast cancer (MBC) that can be explained by initial tumor heterogeneity as well as by evolutionary changes during therapy and tumor progression. Circulating tumor cells (CTCs) detected in a liquid biopsy can be sampled and characterized repeatedly during therapy in order to monitor treatment response and disease progression.

Our aim was to investigate how CTC derived gene expression of treatment predictive markers (*ESR1*/*HER2*) and other cancer associated markers changed in patient blood samples during six months of first-line systemic treatment for MBC. CTCs from 36 patients were enriched using CellSearch (Janssen Diagnostics) and AdnaTest (QIAGEN) before gene expression analysis was performed with a customized gene panel (TATAA Biocenter).

Our results show that antibodies against HER2 and EGFR were valuable to isolate CTCs unidentified by CellSearch and possibly lacking EpCAM expression. Evaluation of patients with clinically different breast cancer subgroups demonstrated that gene expression of treatment predictive markers changed over time. This change was especially prominent for *HER2* expression.

In conclusion, we found that changed gene expression during first-line systemic therapy for MBC could be a possible explanation for treatment resistance. Characterization of CTCs at several time-points during therapy could be informative for treatment selection.

## INTRODUCTION

Tumor heterogeneity poses a large problem for therapeutic strategies in patients with metastatic breast cancer (MBC). Sub-clonal tumor cell populations from the primary tumor or early spread cancer cells are not always eradicated by the selected therapies and these cells might therefore continue to proliferate and metastasize. Circulating tumor cells (CTCs) found in the blood during therapy are possible representatives of these treatment resistant sub-clonal cell populations and provide an opportunity to study the characteristics of minimal residual disease by a simple blood sample – a liquid biopsy.

The prognostic value of CTC number in patients with MBC has been thoroughly investigated [1, 2] but research focus is now turning to the possible treatment predictive value of CTCs. In MBC, treatment resistance inevitably occurs and leads to disease progression and death in the majority of patients. To improve patient survival, it is important to understand how subsets of cancer cells survive initially efficient treatment and how these specific cells can be targeted. It is possible that new and combined treatment strategies can be used to avoid development of resistance and thereby prolong patient survival [3].

The most important treatment predictive markers in breast cancer are Estrogen receptor (ER) and Human epidermal growth factor receptor 2 (HER2). Detection of either of these markers in the primary tumor or metastatic lesions are predictive of endocrine or HER2 targeted therapy, respectively, whereas absence of these markers (combined with absence of Progesterone receptor (PgR)) defines the Triple-negative breast cancer (TNBC) subgroup where chemotherapy is the current standard treatment. The prognosis for patients with TNBC is dismal and ongoing clinical trials are investigating new treatments in addition to chemotherapy [4, 5]. However, it has become widely accepted that the expression of ER, PgR and HER2 frequently changes between primary tumor and metastasis, and that the choice of therapy should be based on the receptor status of the metastatic biopsy when available [6–9]. Multiple biopsies from the same patient have also shown high genetic heterogeneity between different metastatic sites within the same patient [10, 11]. In clinical praxis, however, it is not feasible to biopsy multiple metastatic sites due to cost as well as the increased risk and discomfort for the patient.

CTCs detected in a liquid biopsy could be used for both gene expression analysis and determination of protein expression, in addition to genomic analyses, and the malignant and metastatic nature of CTCs in the circulation has been studied [12–14]. CTCs have also been shown to reflect tumor heterogeneity more accurately than biopsies of a single metastasis, possibly due to representation of different metastatic sites [15–18]. CellSearch (Janssen Diagnostics, Raritan, NJ, USA) is the most established and the only FDA approved detection and isolation method for CTCs. Enumeration of CTCs with CellSearch is a proven independent prognostic factor in MBC as well as in other types of cancer [1, 2]. Detection of CTCs with CellSearch is based on expression of the Epithelial cell adhesion molecule (EpCAM) and cytokeratins (CK) 8, 18 and 19. This limits the detection of the CTCs that have downregulated expression of epithelial markers through for example epithelial-to-mesenchymal transition (EMT) [19–22]. Cancer cells that have gone through EMT have been suggested to represent a more aggressive and metastasis competent population and this is a concern if these cells are not detected with CellSearch [20, 23–25]. A method that has been shown to capture both epithelial and mesenchymal CTCs is AdnaTest (QIAGEN, Hannover GmbH, Germany) [26–28] where CTC capture is based on other markers in addition to EpCAM, such as Mucin 1 (MUC-1), HER2 and Epithelial growth factor receptor (EGFR).

Using different isolation methods for CTC capture, studies have shown that the ER and HER2 status of CTCs are in many cases different to that of the primary tumor [29–35] and/or the metastatic tissue [36]. On the other hand, recent results from the German DETECT Study Group showed that CTC HER2 status could be used to predict HER2 status of metastases and that the variation between primary tumor, CTCs and metastases could be smaller than previously described [37]. Thus, the extent of marker discordance between primary tumor, CTCs and metastases is still under debate and, more importantly, the treatment predictive value of these changes has to be investigated [9, 31, 38, 39].

Further molecular characterization of CTCs can also be used to improve our knowledge of the metastatic process and to identify new treatment predictive markers [40, 41]. It has been found that CTCs from patients with breast cancer have increased expression of EMT-related genes as well as of genes associated with stem-cell like features [26, 27, 42, 43]. Molecular analyses on single cell level have also revealed large intra-patient as well as inter-patient variability when investigating marker expression in CTCs from breast cancer patients [14, 44, 45]. These results suggest that CTCs constitute a heterogenic cell population composed of cancer cells from several different metastatic lesions as well as from occult micro-metastases. So far, few studies have investigated the change in marker expression on CTCs from individual patients during treatment, but early results provide clear indications that molecular changes do occur over time [14, 35, 43–47].

The primary aim of this study was to molecularly characterize CTCs enriched at several time-points from patients with MBC scheduled for first-line systemic therapy. To determine if CTCs could provide clinically important information, we investigated expression changes of the well-known treatment predictive genes *Estrogen receptor 1* (*ESR1*) and *HER2/neu* (*HER2*) during six months of systemic therapy. Another of our aims was to investigate the change in other markers detectable in isolated CTCs that could be of importance for tumor progression, metastasis and treatment resistance. By using two different capturing methods for CTCs (CellSearch and AdnaTest) we investigated if CTCs could be detected in the same patients by both methods or if AdnaTest was indeed able to find more mesenchymal CTCs.

## RESULTS

### Patient cohort

**Figure 1:**
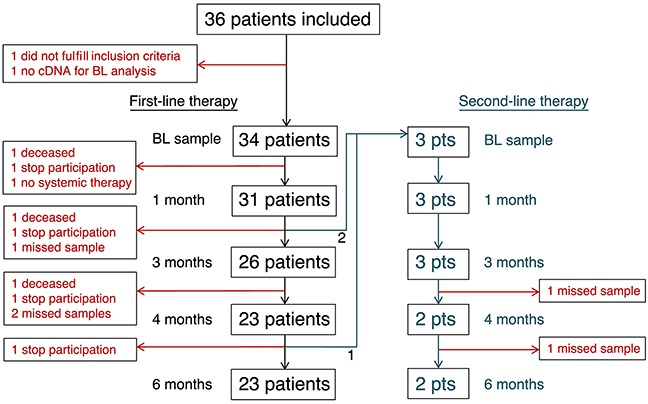
Study design Flow chart of all included patients at the different sample taking time-points in the study. Samples described as “missed sample” were only lost at the subsequent time-point and these patients had not left the study.

A summary of patient and tumor characteristics can be found in Table 1. Thirty-six patients from the ongoing CTC-MBC study (Clinical Trials NCT01322893) were included in the study and 34 of these patients could be evaluated with both CellSearch and AdnaTest (Figure 1). Median follow-up (FU) from base-line (BL) sample taking date to last clinical FU for patients alive at the last review of the records was 14 months (range 7-32 months). The estimated 12-months progression-free survival (PFS) was 58% (95% CI 40-73%). In total, 19 patients had progressive disease (PD) during the study and of these, 11 patients had PD during the first six months of treatment (early PD). The estimated 12-months overall survival (OS) was 72% (95% CI 52-84%) and the total number of deaths in the cohort was 12. Gene expression of CTCs in FU samples was analyzed in 27 patients who had samples available from at least three time-points during the study.

**Table 1:** Patient and tumor characteristics for patients analyzed in the study

	All patients (n=34)	CTC positive patientsa (n=18)	CTC negative patientsb (n=16)	*P*-value
**Age at diagnosis of MBC**				
Median (range) in years	65 (39-83)	66 (45-83)	63 (39-80)	0.63^e^
**Time from initial BC diagnosis to recurrence**				
Median (range) in years	6.5 (0-27)	7 (0-27)	6.5 (0-16)	0.90^e^
**Stage IV at initial diagnosis**				
No	26	12	14	0.23^f^
Yes	8	6	2	
**Breast cancer subtype^c^**				
HR positive	20	11	9	0.28^f^
HER2 positive	6	2	4	
TNBC	6	5	1	
Unknown	2	0	2	
**First-line systemic treatment**				
Endocrine only	12	4	8	0.07^f^
Chemotherapy	16	12	4	
HER2 targeted	5	2	3	
No treatment	1	0	1	
**Metastatic location at BL**				
Loco-regional	5	2	3	0.16^f^
Bone only	10	8	2	
Visceral	16	6	10	
CNS	3	2	1	
**Number of metastatic locations**				
1-2	26	12	14	0.23^f^
≥3	8	6	2	
**CellSearch BL CTC number**				
Median (range)	1 (0-359)	45 (0-359)	0 (0-2)	<0.001^e^
Mean	38	73	0	
**Progressive disease (PD)^d^**				
Yes	19	10	9	0.97^g^
No	15	8	7	
12-months progression-free survival, % (95% CI)	58 (40-73)	61 (35-79)	55 (28-76)	0.60^h^
**Early PD (within 6 months)**				
Yes	11	7	4	0.39^g^
No	23	11	12	
**Death**				
Yes	12	7	5	0.64**^g^**
No	22	11	11	
12-months survival, % (95% CI)	72 (52-84)	78 (51-91)	65 (35-83)	0.55^h^

All factors were compared between CTC positive patients (≥5 CTCs by CellSearch or defined as positive by AdnaTest) and CTC negative patients (<5 CTCs by CellSearch and considered negative by AdnaTest) BL: base-line; CI: confidence interval; CTC: circulating tumor cell; HR: hormone receptor; MBC: metastatic breast cancer; NHG: Nottingham histological grade; OS: overall survival; PD: progressive disease; PFS: progression-free survival; PT: primary tumor; TNBC: triple-negative breast cancer

^a^ CTC positive patients were defined by either ≥5 CTCs identified by the CellSearch system, or the presence of EpCAM, MUC1 or HER2 PCR fragments identified with AdnaTest Breast Cancer Detect.

^b^ CTC negative patients were defined by either <5 CTCs by CellSearch and identified as negative for AdnaTest breast cancer markers as described above.

^c^ Breast cancer subtype was defined as: HR positive (ER+, PgR+/−, HER2-), HER2 positive (ER+/−, PgR+/−, HER2+), TNBC (ER-, PgR-, HER2-). Patients with unknown subtype were not included in the analysis.

^d^ First diagnosis of clinical progressive disease (PD) after inclusion in the study

^e^ Mann-Whitney U-test

^f^ Fisher's exact test

^g^ Pearson Chi-square test

^h^ Log-rank test

Information on breast cancer subtype was available for 32 patients (Table 1): 20 HR+, six HER2+ and the remaining six TNBC. Two patients lacked information on HER2 status and where not classified in a subtype. Information about the patient's diagnostic breast cancer subgroup was based on pathology reports from metastatic tissue in available cases (n=24) or from the primary tumor if metastatic tissue was not available (n=8). Fifteen patients had information available from both metastatic tissue and primary tumor tissue and two of these patients (#8 and 13) experienced a switch in breast cancer subtype from primary tumor (HR+) to metastatic tissue (TNBC). Of the 20 HR+ patients, 10 patients received endocrine therapy and 10 patients received chemotherapy as first-line treatment. Five of the six patients diagnosed with HER2+ disease were treated with HER2 targeted therapy whereas one patient declined systemic therapy and received best supportive care. All six patients with TNBC were treated with chemotherapy. Individual data on treatment and breast cancer subtype for all patients can be found in Table 2.

**Table 2:** Detailed description of all patients included in the study

ID	BL		1 month		3 months		4 months		6 months		PFS (mth)	OS (mth)	FU (mth)	First-line treatment	BC subtype
	CS	Adna	CS	Adna	CS	Adna	CS	Adna	CS	Adna					
1	12	-	14	-	n/a	n/a	0	-	0	-	24		32	Chemo	HR+
3^a^	151	+	57	+	5	-	8	-	0	-	2	28	28	Endocrine	HR+
4	2	-	0	-	0	-	0	-	0	-	9		31	HER2-targ	HER2+
5	45	+	3	-	1	-	1	-	0	n/a			8	Chemo	HR+
6	0	-	n/a	-	0	-	0	-	0	-			28	HER2-targ.	HER2+
7	0	-	1	-	0	-	n/a	-	0	-			21	Chemo	HR+
8	8	+	1	-	2	+	6	+	48	+	4	14	14	Chemo	TNBC
9	0	-	0	-	0	-	0	-	0	-	19		25	HER2-targ.	HER2+
10	1	-	0	-	n/a	n/a	n/a	n/a	n/a	n/a	8	9	9	Endocrine	HR+
11	1	-	0	-	0	-	0	-	3	-	20		24	Endocrine	HR+
12	1	-	0	-	0	-	0	-	0	-	10	11	11	Endocrine	n/a
13	0	-	1	-	0	-	0	-	n/a	n/a	6	11	11	Chemo	TNBC
14	104	-	60	n/a	75	-	n/a	n/a	n/a	n/a	1	6	6	Chemo	TNBC
15	0	-	0	-	0	-	0	-	0	-			18	Endocrine	HR+
16	n/a	+	64	+	n/a	n/a	n/a	n/a	n/a	n/a	1	1	1	Chemo	TNBC
17	0	-	n/a	n/a	n/a	n/a	n/a	n/a	n/a	n/a	1	9	9	Best supportive care	HER2+
18	0	-	0	-	0	-	0	-	0	-			18	Chemo	HR+
20	0	+	0	-	0	-	0	-	0	-			17	Chemo	HR+
21	189	+	0	-	0	-	0	-	0	-			15	HER2-targ.	HER2+
22	33	+	0	-	0	-	n/a	n/a	6	-	13		16	Endocrine	HR+
23	55	+	1	-	1	-	n/a	n/a	37	+	13	16	16	Endocrine	HR+
24^a^	0	-	0	-	n/a	-	0	-	0	-	5		14	Chemo	HR+
25	78	+	3	-	0	-	0	-	1	-			14	Chemo	HR+
26	3	+	0	-	0	-	0	-	0	-			14	HER2-targ.	HER2+
27	14	+	12	+	4	+	2	+	1	+			12	Endocrine	HR+
28	5	-	2	-	1	-	0	-	0	-			13	Chemo	HR+
29	0	-	n/a	n/a	n/a	n/a	n/a	n/a	n/a	n/a	5	5	5	Endocrine	n/a
30	0	-	0	-	0	-	0	-	0	-			10	Endocrine	HR+
31	0	-	0	-	0	-	0	-	0	-			11	Endocrine	HR+
32	1	+	0	-	0	-	0	-	1	-			11	Chemo	HR+
33	359	+	56	-	0	-	n/a	n/a	n/a	n/a	2	4	4	Chemo	TNBC
34	0	-	0	-	0	-	0	-	0	-			9	Endocrine	HR+
35	111	+	n/a	n/a	n/a	n/a	n/a	n/a	n/a	n/a	2	2	2	Chemo	TNBC
36^a^	82	+	45	+	10	+	24	+	34	+	2		9	Chemo	HR+

For each time-point, CTC presence is presented as CS = CellSearch number and Adna = presence of transcripts with AdnaTest Breast Cancer assay. For each patient the number of months for progression-free survival (PFS), overall survival (OS) and follow-up time (FU), as well as first-line treatment and breast cancer (BC) subtype are presented. No number in the PFS and/or OS column displays that the patient is progression-free and/or alive at the last follow-up

Adna: AdnaTest; BC: breast cancer; BL: base-line; Chemo: chemotherapy; CS: CellSearch; HR: hormone receptor; OS: overall survival; PD: progressive disease; PFS: progression-free survival; TNBC: triple-negative breast cancer

^a^Patients with second-line treatment were also followed at closer time-points and for a longer time, but this table demonstrates the CTC results at similar time-points as in the other samples.

**Figure 2:**
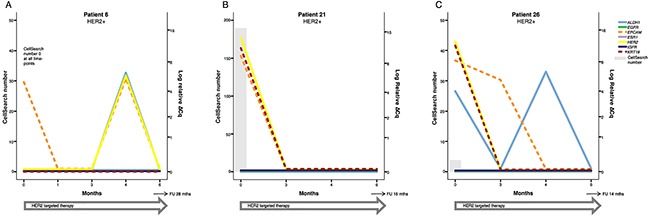
Three examples of patients with HER2 positive breast cancer After initiation of HER2 targeted therapy, none of the patients experienced PD during the study. (**A**) Patient 6 had no detectable CTCs at any time-point but had *EpCAM* expression at BL and at 4 months. Possibly, the lack of *KRT19* expression lead to that CTCs were not detected by CellSearch and AdnaTest despite initial capture. *HER2* expression was also found at 4 months. (**B, C**) Patients 21 and 26 both showed a response to therapy with regard to CTC number and cancer related gene expression in FU samples. *ALDH1* expression continued to vary in patient 26, but this did not seem to affect prognosis. Note that the time course (X-axis) and the scale for CTC number (left Y-axis) varies between patients.

**Figure 3:**
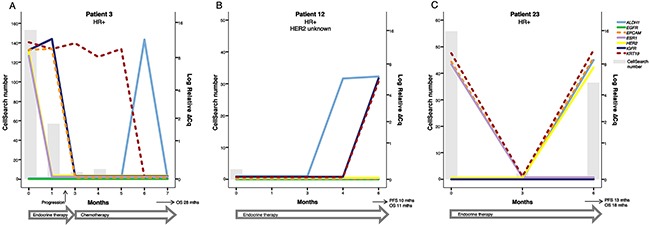
Three examples of patients with HR positive breast cancer (**A**) Patient 3´s treatment was changed from endocrine therapy to second-line chemotherapy after progression at 2 months. Before start of therapy the patient had detectable *ESR1*+ and *HER2*+ CTCs that vanished after treatment initiation. The patient responded well to chemotherapy with an overall survival of 28 months. (**B**) Patient 12 had very few detactable CTCs at BL with the CellSearch method and was CTC negative during therapy. However, at 6 months there was an increase in *KRT19*, *IGF1R* and *ALDH1* expression and the patient was later diagnosed with clinical progression at 10 months. It is possible that this increase in markers at 6 months was an early sign of treatment resistance. (**C**) Patient 23 initially responded to endocrine therapy but the CTC number increased at 6 months and the simultaneous increase in *HER2* expression suggested a phenotype shift in the metastasis that could possibly have been therapeutically targeted. Note that the time course (X-axis) and the scale for CTC number (left Y-axis) varies between patients.

**Figure 4:**
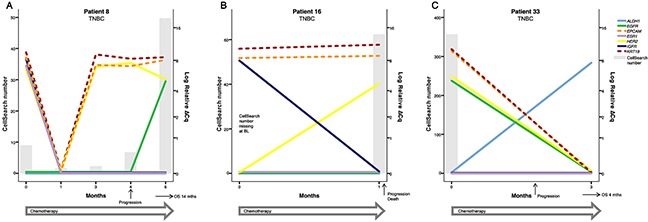
Three examples of patients with TNBC treated with chemotherapy (**A**) Patient 8 had detectable CTCs at BL with both *ESR1* and *HER2* expression despite being clinically diagnosed with TNBC. After an initial response to therapy, the number of *HER2*+ CTCs started to increase at 3 months and the patient was diagnosed with PD after 4 months of treatment. Interestingly, *EGFR* expression was also detectable at 6 months indicating a phenotype change and a possible targetable marker in addition to HER2. (**B**) Patient 16 had a rapid disease progression but the increased *HER2* expression at 1 month could possibly have been targetable. (**C**) Patient 33 also had a rapid progression and despite a decrease in both CTC number and in markers such as *HER2* and *EGFR* after initiation of chemotherapy, the patient quickly experienced disease progression and death. The only marker visible in the blood at 3 months (two days before the patient died) was expression of the stem cell marker *ALDH1*. However, *ALDH1* expression was not correlated to prognosis in this cohort. Note that the time course (X-axis) and the scale for CTC number (left Y-axis) varies between patients.

CTC derived gene expression data from nine individual patients are presented in detail in Figures 2–4. The graphs illustrate how gene expression of *ALDH1*, *EGFR*, *EpCAM*, *ESR1, HER2*, *IGF1R*, and *KRT19* (full names can be found in Table 3 ) fluctuates in patients with HER2+ (Figure 2), HR+ (Figure 3) and TNBC (Figure 4) subtype, respectively, in relation to systemic therapy, PD, death and the number of CTCs detected with CellSearch.

**Table 3:** Included genes in TATAA Biocenter gene panel and the association between BL gene expression of each marker and PFS, OS and early PD. 38 breast cancer related genes and nine reference genes were included in the analysis

Gene	Full name	Number of patients with expression at BL		PFS (*P*-value)	Early PD (*P*-value)	OS (*P*-value)	Protein function
		Yes	No				
*AKT2*	V-akt murine thymoma viral oncogene homolog 2	22	12	0.34	0.14	0.88	ER, EMT
*ALDH1*	Aldehyde dehydrogenase 1 family, member A1	8	26	0.65	0.21	0.25	SC, TR
*CCND1*	Cyclin D1	13	21	0.16	0.15	0.20	P
*CD24L4*	CD24 molecule	Excluded (technical problems)					
*CD44*^a^	CD44 molecule	31	3	0.53	0.53	0.15	SC
*CDH1*	Cadherin 1, type 1, E-cadherin	8	26	0.10	0.18	0.40	Ep
*CTSD*^a^	Cathepsin D	31	3	0.76	0.66	0.80	M, A
*EGFR*^a^	Epidermal growth factor receptor	1	33	0.27	0.21	0.09	P, ER, TR
*EpCAM*	Epithelial cell adhesion molecule	15	19	0.43	0.36	0.20	Ep
*ESR1*^a^	Estrogen receptor 1	4	30	0.09	0.48	0.37	P
*FOXO*	Forkhead box O3	18	16	0.59	0.94	0.84	ER
*HDAC2*	Histone deacetylase 2	29	5	0.28	0.09	0.14	M, EMT
*HER2*	Human epidermal growth factor receptor 2	10	24	0.25	0.51	0.24	P, TR
*IBSP1*	Integrin-binding sialoprotein	Excluded (technical problems)					
*IGF1R*^a^	Insulin-like growth factor 1 receptor	3	31	0.03	0.01	0.04	P, ER
*Ki67*^a^	Antigen identified by monoclonal antibody Ki-67	1	33	0.53	1.0	0.79	P
*KI*^a^	V-kit Hardy-Zuckerman 4 feline sarcoma viral oncogene homolog	1	33	0.26	0.20	0.08	P
*KRAS*	V-Ki-ras2 Kirsten rat sarcoma viral oncogene homolog	Excluded (technical problems)					
*KRT19*	Keratin 19	20	14	0.01	0.05	0.06	Ep
*MET*	Met proto-oncogene	No expression					
*mTOR*	Mechanistic target of rapamycin	25	9	0.26	0.36	0.12	ER
*MUC1*^a^	Mucin 1	3	31	0.88	0.91	0.69	Ep
*Myc*	V-myc avian myelocytomatosis viral oncogene homolog	25	9	0.89	0.42	0.72	P, ER, TR
*PARP*	Poly (ADP-ribose) polymerase 1	28	6	0.88	0.99	0.34	P
*PGR*	Progesterone receptor	No expression					
*PI3KCA*	Phosphoinositide-3-kinase, catalytic, alpha polypeptide	15	19	0.07	0.17	0.04	ER, EMT
*PTEN*	Phosphatase and tensin homolog	29	5	0.54	0.19	0.32	ER
*RAD51*	RAD51 recombinase	9	25	0.32	0.44	0.69	M
*SATB1*	SATB homeobox 1	17	17	0.70	0.75	0.30	M
*SCGB2A/MAM*	Secretoglobin, family 2A, member 2	7	27	0.91	0.49	0.65	M
*TOP2A*	Topoisomerase (DNA) II alpha 170kDa	12	22	0.88	0.94	0.85	TR
*TP53*	Tumor protein p53	23	11	0.81	0.73	0.60	P, TR
*Twist1*	Twist family bHLH transcription factor 1	Excluded (technical problems)					
*UPA*^a^	Plasminogen activator, urokinase	2	32	0.11	0.06	0.28	M
*VEGFA*	Vascular endothelial growth factor A	6	28	0.55	0.97	0.72	A
*VEGFR1*	Vascular endothelial growth factor receptor 1, fms-related tyrosine kinase 1	0	34	-	-	-	A
*VEGFR2*^a^	Vascular endothelial growth factor receptor 2, kinase insert domain receptor	2	32	0.96	0.55	0.61	A
*VIM*^a^	Homo sapiens vimentin	31	3	0.59	0.66	0.57	EMT
*ACTB*	Beta actin	Reference gene^b^					
*B2M*	Beta-2-microglobulin	Reference gene					
*GAPDH*	Glyceraldehyde-3-phosphate dehydrogenase	Reference gene					
*HPRT1*	Hypoxanthine phosphoribosyltransferase 1	Reference gene					
*PPIA*	Peptidylprolyl isomerase A (cyclophilin A)	Reference gene					
*RPLP*	Ribosomal protein, large, P0	Reference gene^b^					
*TBP*	TATA box binding protein	Reference gene					
*UBC*	Ubiquitin C	Reference gene					
*YWHAZ*	Tyrosine 3-monooxygenase/tryptophan 5-monooxygenase activation protein, zeta polypeptide	Reference gene					

BL expression of each marker was analyzed against PFS, early PD (within 6 months) and OS. Genes were divided into different groups by protein function in cancer: P: proliferation/growth/survival, M: metastasis/migration, A: angiogenesis, Ep: epithelial marker, EMT: EMT marker, SC: stem cell marker, ER: endocrine therapy resistance, TR: treatment resistance (chemotherapy, HER2 targeted therapy)

^a^Genes for which <5 patients were classified in one group were analyzed with a permutation based log-rank test.

^b^Reference genes selected for further analysis using NormFinder algorithm [79].

### CTC presence by CellSearch and AdnaTest

Blood samples were collected from each patient before start of therapy (BL) and after approximately 1, 3, 4, and 6 months of first-line therapy. In three patients, treatment was changed during this time-course and new samples were taken at corresponding time-points (BL, 1, 3, 4, and 6 months) during second-line treatment (Figure 1). At each time-point, 7.5 ml blood was analyzed by CellSearch and 2 × 5 ml blood by AdnaTest EMT1 and EMT2 kits, respectively. Patient and tumor characteristics in relation to the presence of CTCs defined by either CellSearch or AdnaTest are presented in Table 1. Eighteen of 34 patients (53%) were positive for CTCs with at least one of the methods (according to the manufacturer´s instructions), and these patients are referred to as “CTC positive” throughout the rest of the paper. All 18 patients were positive at BL and 10 patients were also positive at one or more later time-points (Table 2).

At BL, 14 of 33 patients (42%) were CTC positive as defined by CellSearch with ≥ 5 CTC/7.5 ml blood (Table 2). For one patient a CellSearch BL sample was missing due to technical issues. The AdnaTest Breast Cancer assay identified 15 of 34 CTC positive patients (44%) at BL (PCR based detection of *EpCAM*, *HER2* or *MUC1* transcript ≥ 15ng/ul). However, six patients were identified as CTC positive by only one of the applied methods, three patients with only CellSearch and three patients with only AdnaTest. Interestingly, CTCs in all discordant cases identified as positive exclusively with AdnaTest, were only detected by the EMT2 kit (isolation based on EpCAM, HER2, and EGFR protein expression). Agreement between the two methods regarding CTC positivity at BL was found in 28 of 33 patients (85%, Table 2 ). When comparing all samples (n=129) with data from both AdnaTest and CellSearch, agreement was found in 115 of 129 samples (89%), which can be classified as good (κ=0.68).

Patients with CTCs present were more often treated with chemotherapy compared to the group with no CTCs present (Table 1). In the latter group, a majority of patients received only endocrine treatment. This difference was partly due to a higher number of patients with TNBC found in the group of CTC positive patients. Indeed, five of six patients with TNBC had detectable CTCs when the BL sample was taken (Table 1).

### Gene panel and heatmap

**Figure 5:**
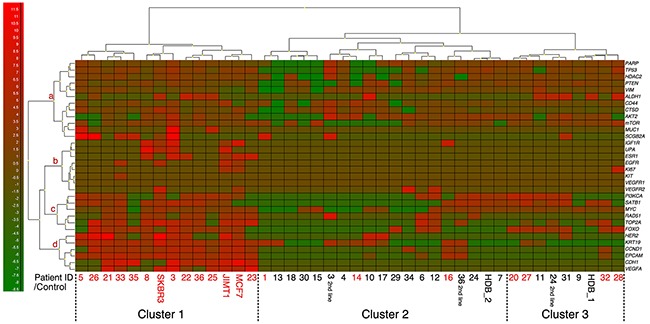
Mean-centered heatmap showing the expression of 32 genes detectable in at least one of the BL samples Three distinct patient clusters denoted 1, 2 and 3 were found with all cell line cells (positive controls) in cluster 1 and healthy donor blood (negative controls) in clusters 2 and 3. Patient ID marked with red were CTC positive by either CellSearch or AdnaTest. Genes were clustered in four groups (a-d) where group d included the most commonly used markers for CTC positivity. Genes with very low expression could be found in group b. The highest expression of CTCs captured with the EMT1 or EMT2 kit is shown.

**Figure 6:**
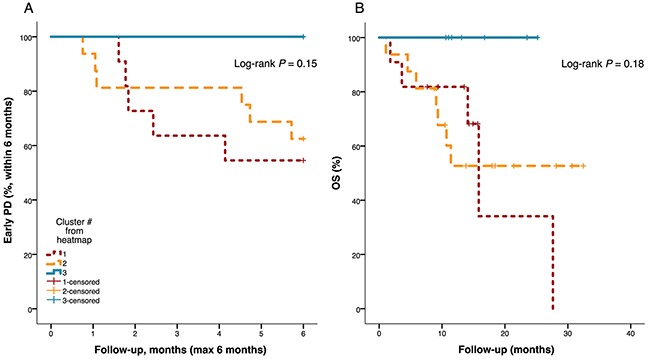
Kaplan-Meier plots for progression-free and overall survival by heatmap patient cluster No patients from heatmap cluster 3 experienced disease progression within 6 months (**A**) or died during the course of the study (**B**). *P*-values from log-rank test.

In addition to CTC analyses by CellSearch and the AdnaTest Breast Cancer assay, enriched samples from AdnaTest EMT1 and EMT2 based isolation were analyzed by multiplex qPCR for 38 genes associated with cancer. In total, 32 of the 38 included experimental genes could be analyzed in the final cohort. Four markers had to be excluded due to technical problems and two showed no expression in any sample (Table 3). A heatmap of all BL samples together with positive and negative controls can be found in Figure 5. The three cell lines SKBR3, MCF7 and JIMT1 were included as positive controls and two different healthy donor blood (HDB1 and 2) were included as negative controls to add information on leukocyte derived gene expression. Based on expression of the included genes, three patient clusters (Clusters 1, 2, and 3 in Figure 5) were clearly visible and all cell line samples were found in cluster 1 together with patient samples that were classified as CTC positive with either AdnaTest or CellSearch (marked in red). HDB samples could be found in clusters 2 and 3 together with both CTC positive and CTC negative patient samples. All patients in cluster 1 were classified as CTC positive and more patients with stage IV disease at initial diagnosis could be found in this cluster (*P*=0.02). Interestingly, none of the seven patients with first-line BL sample in cluster 3 experienced early PD (within 6 months) and all patients in this cluster were alive at the end of the study despite the fact that four of seven patients were classified as CTC positive (Figure 6). Six of seven patients in cluster 3 had HR+ MBC and of these, only one patient experienced PD during the study. This can be compared with four of six patients with HR+ MBC in cluster 1 experiencing PD. Patients were found to switch between different clusters when samples were taken at later time-points during the study. We hypothesized that frequent switching between different clusters would suggest higher plasticity and could be predictive of worse survival. However, this could not be found in the present cohort (data not shown).

The included genes were hierarchically assembled in four groups (a-d). The eight genes found in group b were expressed at low levels at BL (Figure 5) and also in FU samples. Group d included genes for epithelial markers that are used for conventional analyses of CTCs and these genes were important for defining patient cluster 1. Groups a and c were less defined and contained genes of importance in different functional pathways. Generally, all groups of genes had lowest expression in patient cluster 2 (Figure 5).

### Gene expression of conventional CTC markers

**Figure 7:**
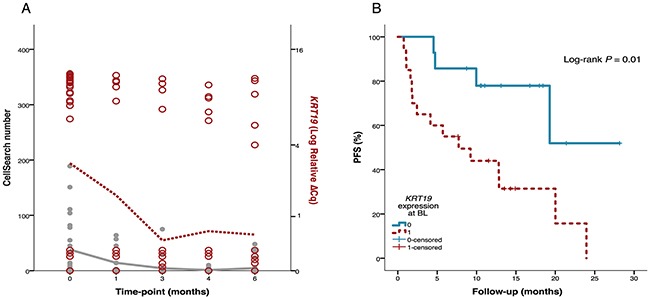
Association of *KRT19* gene expression to CTC number and progression-free survival (PFS) (**A**) Of the conventionally used CTC markers, *KRT19* gene expression (red circles, right axis) showed highest correlation to CTC number measured by CellSearch (grey filled circles, left axis) over time (R_S_ = 0.61). The red dotted line illustrates median *KRT19* expression and the gray solid line illustrates median CTC number at the respective timepoints. (**B**) *KRT19* expression in CTC blood samples taken before start of therapy (BL) was associated with shorter PFS.

The most commonly used markers for CTC positivity are EpCAM, KRT19 and MUC1, and protein expression or PCR products of these markers were also used for classifying CTC positive and CTC negative patients in the present study (Table 1). Gene expression of *EpCAM*/*KRT19*/*MUC1* was analyzed within the gene panel in parallel with CellSearch and AdnaTest. As expected, *EpCAM* and *KRT19* expression at BL were associated with CTC positivity (*P*<0.001 and *P*=0.002, respectively) and *KRT19* expression showed the highest correlation to CTC number over time (R_S_=0.61, Figure 7A). Patients with *KRT19* expression at BL had shorter PFS (*P*=0.01, Figure 7B) and OS (*P*=0.06) and a higher number of patients in this group experienced early PD (*P*=0.05, Table 3 ). The evidence for association to these outcomes was weaker for *EpCAM* expression. *MUC1* was only expressed at BL in three patients in the whole cohort. All of these patients were ER+ and classified as CTC positive. There was no association to PFS or OS.

E-cadherin (*CDH1*) is another epithelial marker but it is not as commonly used for CTC detection as the above mentioned. *CDH1* expression was found in eight patients at BL and all of these patients also expressed *EpCAM* and *KRT19*. *CDH1* expression was associated with CTC positivity (*P*=0.003). However, the conventional markers were expressed in a higher number of patients (*EpCAM* n=15, *KRT19* n=20) than *CDH1* (n=8, Table 3).

### *HER2* expression and the HER2 positive subgroup

Six patients in this cohort were, according to the pathology report, diagnosed with HER2+ MBC. All patients that received HER2 targeted therapy responded well (Table 2). This was also reflected in rapid CTC clearance after treatment initiation (Figure 2).

Gene expression of *HER2* in CTCs did not reflect the clinical diagnosis of HER2 status either at BL or in FU samples. At BL, *HER2* was expressed only in three of six patients with clinical HER2+ disease but this could be due to lack of CTCs in the other three patients. However, *HER2* was expressed at BL in five of 10 patients with HR+ breast cancer (e.g. Figure 3A) and in two of six patients with TNBC (Figure 4A and 4C). In FU samples, nine of 27 evaluable patients expressed *HER2* and of these, only one patient was clinically diagnosed with HER2+ breast cancer and treated with HER2 targeted therapy (#6, Figure 2A). Seven of the nine patients were clinically diagnosed with HR+ disease (#1, 22, 23 (Figure 3C), 27, 31, 32, and 34) and one of the nine with TNBC (#8, Figure 4A), and they received either endocrine treatment or chemotherapy (Table 2). Patient #16, with very rapid progression of TNBC, had *HER2+* CTCs at 1 month and died shortly after identification of *HER2* expressing CTCs (Figure 4B). In the whole cohort, the expression of *HER2* at FU time-points was not found to be associated to PD or death.

### *ESR1* expression and resistance against endocrine treatment

*ESR1* (ER) expression was only found in four BL samples and not in any FU samples after initiation of endocrine treatment (#3, 22, and 23) or chemotherapy (#8), see Figures 3 and 4 for examples. Three of the four patients with BL samples that expressed *ESR1* before start of therapy had clinically diagnosed HR+ breast cancer but in one patient (#8) the disease had changed phenotype from primary tumor (HR+) to metastasis (TNBC, Table 2). The total number of patients with clinically HR+ disease that also had CTCs detected with conventional markers by CellSearch or AdnaTest was nine. Thus, our results indicate that at least 67% (6/9) of the originally HR+ patients were devoid of *ESR1* expression on CTCs already before start of therapy.

Expression of genes involved in endocrine resistance (Table 3) were investigated in the subgroup of HR+ patients treated with endocrine treatment and cytotoxic therapy, respectively. The expression patterns of these genes were not different between patients that received either type of therapy (data not shown). Also, the expression pattern was not different between endocrine treated patients that had PD versus non-PD. A few of the investigated genes have previously been associated with chemotherapy resistance (Table 3) but no difference in expression pattern was found for these genes between patients that had PD versus non-PD.

As mentioned above, *HER2* was expressed in a high proportion of CTCs in patients with HR+ breast cancer treated with endocrine therapy. In patients with HR+ breast cancer, *HER2* gene expression was more common in patients that received endocrine treatment (5/9) compared to patients that received chemotherapy (2/10; *P*=0.17). In patient #3, *HER2+* CTCs were present at BL and this patient did not respond to first line endocrine treatment (Figure 3A). Acquired *HER2+* CTCs were detected in for example patient #23 in this cohort (Figure 3C).

### Other expressed markers of interest

The expression of all markers at BL was analyzed against clinico-pathological characteristics as described in the method. Interesting findings are reported here (see also Table 3). Weak to moderate association with CTC positivity (detected by CellSearch or AdnaTest) at BL was found for: *FOXO* (*P*=0.02), *mTOR* (*P*=0.05), *SATB1* (*P*=0.006), *TOP2A* (*P*=0.009), and *TP53* (*P*=0.005). *TP53* expression was also positively associated with having stage IV disease at initial diagnosis (*P*=0.02). Of the six patients that expressed *VEGFA* at BL, five had only bone metastases (*P*=0.03). Expression of *PI3KCA* was associated with improved overall survival (Table 3).

*Insulin-like growth factor 1 receptor* (*IGF1R*) was expressed in seven patients (eight samples) and was in all cases expressed only in EMT2 captured samples. *IGF1R* expression showed no association with CTC positivity but was expressed in patients with CTCs detected with conventional markers as well as in patients with no CTCs detected. *IGF1R* was expressed in all investigated cell lines but also in one HDB sample. Thus, it is possible that *IGF1R* expression could be derived from non-cancer cells. However, six of the seven patients that expressed *IGF1R* at any time-point during the study experienced PD and four of seven experienced early PD. Only three patients expressed *IGF1R* in BL samples (#3, 8 and 16) and, recognizing the small number of positive samples, this expression was associated with early PD and shorter OS (Table 3), see examples in Figures 3A and 4A.

Only two samples expressed *Epidermal growth factor receptor* (*EGFR*), patient #8 at six months and patient #33 at BL (Figure 4A and 4C). Both of these patients experienced PD within six months, in close proximity to detected *EGFR* gene expression. *EGFR* was consistently expressed in all cell line cells but in no HDB samples, suggesting that *EGFR* gene expression was derived from CTCs. Both *EGFR* positive patients were also CTC positive at BL and at the time of *EGFR* detection. Since both patients were diagnosed with TNBC they were likely to experience early PD and death but the upregulation of *EGFR* expression in patient #8 after 6 months of chemotherapy clearly shows that cancer cells survive and continue to evolve during chemotherapy (Figure 4A).

The stem-cell marker *Aldehyde dehydrogenase 1* (*ALDH1*) was expressed in eight of 34 (24%) BL samples and not correlated to outcome or any other factor at this time-point. At FU, *ALDH1* expression was found in 15 of 27 (56%) of the patients and only weak evidence was found for association to PD for patients with *ALDH1* expression in their FU samples (*P*=0.07). In the subgroup of patients with TNBC, only one patient (#33, Figure 4C) expressed *ALDH1* at one time-point and this could have been suggestive of association with progression. However, *ALDH1* expression was found also in patients irrespective of CTC number or progression (#12, Figure 3B and #26, Figure 2C) as well as in one of the HDB samples. A majority of FU *ALDH1* positive patients had visceral metastases (10/15) compared with FU *ALDH1* negative patients (3/12) (*P*=0.16).

Of the five genes associated with EMT included in the gene panel (Table 3), four markers (*AKT2*, *PI3Kα*, *HDAC2*, and *VIM*) were possible to evaluate but neither of these markers showed any association to progression nor survival. Neither did they show a specific gene expression pattern over time as a response to treatment or depending on BC subgroup (data not shown).

Metastasis associated markers (Table 3) were investigated in relation to the number and location of metastases. Patients with high combined expression of *CTSD*, *RAD51*, *SATB1*, and *MAM* over the whole study period were identified and patients (50%) with the highest average expression of these markers had three or more metastatic locations more often than patients with lower average expression (6/8 compared with 12/26, *P*=0.23).

*VEGFA*, *VEGFR1*, and *VEGFR2* are genes of importance for angiogenesis. Detection of *VEGFR1* and *2* gene expression was very rare in this cohort but increased *VEGFA* expression at BL were found exclusively in patient cluster 1, including all cell lines (Figure 5).

## DISCUSSION

In this study, gene expression analysis of CTCs clearly demonstrated a continuous evolutionary change in gene expression during systemic therapy for MBC. Notably, we found that only a subset of patients with clinically diagnosed HR+ breast cancer expressed *ESR1* in CTCs and *ESR1* expression was in all cases absent after initiation of endocrine treatment or chemotherapy. This is in line with previous studies that have reported a low level of ER/*ESR1* expression in CTCs compared with the primary tumor in MBC [29, 30, 48, 49]. It is also worth noting that the absence of *ESR1* expression in FU samples from initially *ESR1* positive cases was not associated with the absence of CTCs (e.g. Patients #3 and 23 (Figure 3A and 3C) and patient #8 (Figure 4A)). CTCs detected by conventional markers using CellSearch or AdnaTest were found also at later time-points during therapy but then expressed genes of other markers. Previous studies have found that downregulation of *ER* is associated with EMT and increased migration as well as invasion [50, 51]. The low *ESR1* expression in CTCs could thus be related to the migratory phenotype of CTCs. It is possible that ER expression is upregulated again in the metastases as discussed by Aktas *et al*. [37] and that targeting potentially metastasis competent cancer cells in the blood stream might be difficult using ER directed therapy.

In contrast to *ESR1* only being expressed in CTCs of very few patients, *HER2* gene expression at BL was found in seven out of 26 patients that were clinically diagnosed with HR+ breast cancer (e.g. Patient #3, Figure 3A) or TNBC (Patient #8 and 33, Figure 4A and 4C). In addition, *HER2* was expressed in three out of six patients with HER2+ disease (Patient #21 and 26, Figure 2B and 2C). A high number of patients displayed *HER2* gene expression after one or several months of either endocrine treatment or chemotherapy. This is interesting since an up-regulation of HER2 expression is known to be an important part of endocrine resistance [52, 53]. One example is Patient #23 (Figure 3C) who was clinically diagnosed with HR+ breast cancer and positive for *ESR1* gene expression at BL. After initiation of endocrine treatment, no CTCs or other cancer associated gene expression could be detected in the blood after three months. However, after six months of therapy, CTCs could again be detected in the blood but at this time-point *ESR1* gene expression was no longer detectable. On the other hand the CTCs now expressed *HER2*, suggesting treatment resistance due to an evolutionary change in gene expression in cancer cells escaping treatment. This patient showed no signs of clinical progression until 12 months after BL and she died three months after this. One could speculate that addition of HER2 targeted treatment at six months could possibly have eradicated this new sub-clonal expansion of cancer cells and contributed to a longer survival time for the patient. A previous proof-of-principle study has shown that three of four clinically diagnosed HER2 negative patients with HER2 positive CTCs responded to HER2 targeted therapy [31] and several ongoing prospective studies investigate this in larger cohorts [38].

Continuous evolutionary changes of *HER2* gene expression were also seen in several patients with TNBC (Figure 4). Patient #8 had CTC *HER2* gene expression both before and during therapy but was only treated with chemotherapy due to clinically diagnosed TNBC. Again, given the gene expression data gained from subsequent CTC sampling it is tempting to speculate that the patient would have benefited from HER2 targeted therapy. Patients #16 and 33 (Figure 4B and 4C) also showed CTC *HER2* expression despite clinically diagnosed TNBC. Both these patients had very rapid disease progression but in the whole cohort, *HER2* gene expression was not associated with worse survival. However, the limited FU time and small sample size must be accounted for. In a larger study of 62 patients with MBC, Bredemeier *et al*. [43] found that CTC derived *HER*2 gene expression increased during therapy in patients not responding to the administered therapy. It has been suggested that HER2+ CTCs could be part of a resistance mechanism since HER2 over-expression is a well-known factor in chemotherapy resistance [54] as well as in resistance to endocrine therapy [52].

In Swedish clinical praxis, ER status is evaluated as protein expression by immunohistochemistry (IHC) whereas HER2 status is evaluated by IHC complemented with FISH/CISH (gene amplification) in ambiguous cases. Thus, according to Swedish national guidelines [55], determination of gene expression by mRNA analysis is not performed in the clinical setting. Gene expression is not always correlated to protein expression but breast cancer subgroups defined by gene expression have been found to be highly correlated to outcome and treatment response, as well as to surrogate molecular subtypes defined by the St Gallen classification [56, 57]. The possibility to follow gene expression of treatment predictive markers over time has been suggested to be one of the major advantages of the liquid biopsy [10, 58–60].

One of the challenges of CTC based methods is the low number of detectable cells using currently available methods. In this study, we have used the commercial and standardized methods CellSearch (Janssen Diagnostics) and AdnaTest (QIAGEN) to enrich CTCs from whole blood. CellSearch employ EpCAM and CK 8, 18 and 19-protein expression to isolate and identify CTCs and this method has gained the highest level of clinical validity in MBC [1]. AdnaTest CTC detection is based on a higher number of markers (EMT1: EpCAM and MUC-1 / EMT2: EpCAM, HER2, EGFR) as an attempt to capture more CTCs of mesenchymal or stem-cell like phenotype [43]. We found high concordance in CTC positivity between the two methods but, interestingly, all three samples that were CTC negative with CellSearch while CTC positive with AdnaTest had detectable CTCs only with the EMT2 kit. This confirms that EGFR and HER2 are useful for CTC capture, as previously shown in a smaller cohort comprised of a mixture of patients with either early or metastatic breast cancer [61]. On the other hand, three samples were CTC positive only with CellSearch, so no method was found to be superior at detecting CTCs in a higher proportion of MBC patients. Thus, we decided to use a combined criterion for CTC positivity, e.g. CTC positivity was defined as CTC positive by either CellSearch or AdnaTest. Interestingly, we found that BL *KRT19* expression in the gene panel analysis predicted progressive disease and death of patients better than CTC presence by either CellSearch, AdnaTest or combined (Table 3, Figure 7). *KRT19* is commonly used as a marker for CTC presence and has frequently been used for CTC detection by molecular qPCR based methods [59, 62, 63].

Gene expression data in this study has been obtained from a mixture of CTCs and leukocytes present in the sample after AdnaTest CTC enrichment. Thus, part of the detected gene expression could be a result from leukocytes present in the patient's circulation and not from CTCs. We have included three different cell lines as positive controls together with two different healthy donor blood (HDB) samples as negative controls. Results from markers that are expressed in HDB as well as in CTCs are interpreted with care since they could come from leukocyte contamination. In a previous study of CTC gene expression, Sieuwerts *et al*. [29] have suggested that it is possible to differentiate between gene expression from leukocytes and CTCs by using *KRT19* gene expression as a control for CTC derived expression. But in contrast to this and many other studies on CTC gene expression, we have also analyzed gene expression from samples where no CTCs could be detected with conventional markers. We consider it likely that CTCs are present in a majority of patients with metastatic breast cancer but that these cells are not isolated and identified with available methods. It is known that cancer cells can be detected in the circulation in a higher proportion of patients using other methods than CellSearch and AdnaTest but the prognostic value of these techniques is still under investigation [64]. Indeed, in this study we found gene expression of *KRT19* in samples with very low or absent numbers of CTCs as detected with CellSearch or AdnaTest (e.g. Patient #3, 8, and 26, Figures 2–4). Also, *HER2* expression was detected in a patient with HER2+ MBC but with no CTCs according to CellSearch or AdnaTest (Patient #6, Figure 2A).

There are several possible explanations for this gene expression in “CTC negative” samples. 1) Expression of potential cancer associated markers could be found in CTCs captured but not detected by CellSearch or AdnaTest (i.e. negative for CK8/18/19, HER2, MUC1 and/or EpCAM), but also in 2) CTCs captured non-specifically as bystanders. 3) Higher sensitivity of detection of for example *HER2* expression in qPCR gene panel analysis than in the PCR based AdnaTest Breast Cancer assay could lead to positive expression in “CTC negative” samples defined by commercial methods. However, as described above, 4) a CTC enriched sample also contains contaminating blood cells and the possibility of leukocyte derived expression of markers has to be taken into account.

We hypothesized that expression of EMT or stem-cell markers such as *ALDH1* could be expressed in samples with no CTCs detected by conventional markers [65], as exemplified in Patient #33 (Figure 4C). At time of progression, this patient had lost expression of conventional CTC markers but we saw an increase in *ALDH1* gene expression. This could possibly be a sign of treatment resistance and/or an expansion of sub-clonal stem-cell like CTCs. *ALDH1* gene expression has in previous studies been used as a marker for stem-like CTCs and is suggested to characterize a more aggressive population of cancer cells that might be associated with therapy failure [26, 27, 66]. However, we found *ALDH1* gene expression also in one HDB sample and in many patient samples without association to disease progression (e.g. Patient #3, 6 and 26, Figures 2–3). High *ALDH1* expression in primary tumors is a general predictor of poor prognosis, early metastasis and progression in breast cancer but the exact mechanism of ALDH1 and its importance in treatment resistance remains to be determined [67].

A variable expression pattern over time was also observed for several other markers in this study, in line with previous observations of variation in CTC gene expression levels during treatment [14]. Thus, it seems plausible that gene expression detected in the primary tumor is not representative of the heterogeneous disease presented in treatment resistant clones emerging after several cycles of systemic therapy. The recurrent phenotype switches between primary tumor and metastases [6, 7] as well as variable expression of stem-cell and EMT markers in CTCs as a response to systemic treatment [14] also suggests phenotypic changes in cancer cells over time. There are several biological models for evolutionary changes in cancer cells over time which can be used to understand cancer progression and development of treatment resistance [68]. First, intratumoral heterogeneity in the primary tumor accounts for resistant phenotypes present prior to treatment initiation and disease detection, but the resistant cells might be very few (sub-clonal) and thus not be detected in clinical analyses [69]. Second, the body's natural defense mechanisms will eradicate many cancer cells due to detection of an abnormal phenotype, changed gene expression, mutations or epigenetic factors. Only a selection of cancer cells survive and contribute to tumor progression. Consequently, cells with metastatic abilities are likely to be different from the cells comprising the bulk of the primary tumor [70]. Third, by applying systemic treatment directed towards the phenotype of the primary tumor and/or metastasis, we therapeutically increase the selective pressure on the cancer cells. For example, therapy directed against ER will target ER+ cells but not cells devoid of ER expression, causing relapse or new metastases more likely to be ER- [71, 72]. Fourth, the microenvironment will affect tumor cells in different metastatic niches in unique ways and thus contribute to heterogeneity between separate metastatic sites. Taken together, the most tumorigenic, therapy resistant and well-adapted cancer cells are the ones likely to survive and multiply during tumor progression and therapy.

One aim of this study was to explore possible new targetable markers. EGFR is a growth factor of great importance for prognosis and treatment in other types of cancer. EGFR protein expression has previously been detected in CTCs in early breast cancer [41] and *EGFR* gene expression was also found in CTCs in non-responders during treatment for MBC [43]. We detected *EGFR* gene expression in two patients with TNBC in close proximity to clinical progression (#8 and 33, Figure 4A and 4C). It is possible that upregulation of *EGFR* give cancer cells new survival mechanisms that are clinically actionable. For example, the HER2 targeting drug *Lapatinib* also targets EGFR and could thus be considered beneficial in the two patients who expressed both *HER2* and *EGFR* in close proximity to progression. Also, EGFR tyrosine kinase inhibitors (TKIs) are under development and clinically used in for example lung cancer treatment [3]. This suggests that the characterization of CTC gene expression can provide new treatment targets, but due to the limited sample size in our study, we mainly investigated markers of known importance for treatment resistance. Also, absence of gene expression in this study does not necessarily describe low expression but could also be due to the absence of detected CTCs where no CTCs were detected with CellSearch or AdnaTest. The design of this study made it possible to investigate cancer associated gene expression also in patients where no CTCs were detected with conventional markers, but as a consequence we focused the analyses on positive gene expression.

Therapy resistance is a major problem in metastatic breast cancer and markers of importance for endocrine resistance (*Akt2*, *FOXO*, *mTOR*, *Myc*, *PI3KCA*, and *PTEN*) were investigated in patients with HR+ breast cancer. No specific gene expression pattern could be detected in association to progression for these patients whether they were treated with endocrine therapy or treated with chemotherapy. However, six of 20 patients with HR+ MBC were found in cluster 3 in the heatmap analysis and these patients were found to have a beneficial prognosis compared to patients in cluster 1 and 2 (Figures 5 and 6). *IGF1R* gene expression is closely linked to *ESR1* expression and has been suggested to play a role in resistance to both endocrine and HER2 targeted treatment [52, 73]. In this cohort, BL *IGF1R* gene expression was found in three patients and was associated with shorter survival and earlier progression. We also found *IGF1R* expression in one HDB sample suggesting that the *IGF1R* expression could possibly come from non-cancer cells. Interestingly, *IGF1R* expression was found only in samples enriched with the AdnaTest EMT2 kit, i.e. with HER2 or EGFR directed antibodies. This indicates that *IGF1R* might not be expressed in CTCs defined by conventional CTC markers and is possibly a sign of a more aggressive phenotype. It also demonstrates the importance of capturing CTCs with more markers than EpCAM. Previous studies have detected IGF1R protein expression in CTCs and suggested that this expression could be a useful marker in clinical trials [74, 75]. However, the role of IGF1R in breast cancer, as well as the complicated interplay between other factors in treatment resistance, is far from understood.

In conclusion, changes in *ESR1* and *HER2* expression from primary tumor to metastasis have clearly established that breast cancer is an evolving disease and, not surprisingly, the use of CTCs to monitor treatment response on a molecular level has gained increased attention during the last few years. Our results clearly show an evolutionary change of gene expression detectable in CTCs in the treatment predictive genes *ESR1* and *HER2* during tumor progression and selection pressure by systemic therapy. We found marker discordance both between primary tumors, metastases and CTCs, as well as between CTCs from the same patient over time. This discordance could be targetable as the treatment is traditionally based on the clinical diagnosis before start of therapy and repeated biopsies are rarely taken during treatment. Several clinical trials are currently investigating if HER2+ CTCs are treatment predictive for HER2 targeted treatment response [38]. However, our data show that repeated blood samples can detect evolutionary changes in gene expression as a response to treatment and tumor progression, and CTC characterization could be a future option to monitor changes during treatment. By molecularly characterizing CTCs surviving first-line treatment for MBC, we could increase our knowledge of treatment resistance. It is tempting to speculate that it will be possible to direct future treatment against these specific cells to avoid progression of treatment resistant populations of cancer cells.

## MATERIALS AND METHODS

### Patient material and controls

Patients with metastatic breast cancer have been included in the ongoing CTC-MBC study (Clinical Trials NCT01322893) at Lund University, Sweden, since 2011. The study aims to investigate how CTC number and characteristics change from treatment initiation (BL) until 6 months of first-line treatment. If patients switch to second-line therapy they are offered to continue the study during this therapy. A total number of 36 patients from the CTC-MBC study were included in the present cohort and 34 of these patients were evaluable (Figure 1). An overview of patient and tumor characteristics can be found in Table 1. Patients were divided into three breast cancer subgroups based on protein expression of hormone receptors (ER and PgR) and HER2 [76]. The information was collected from clinical analysis of metastatic tissue in all available cases (N=24) and from the primary tumor in patients without available metastatic tissue (N=8). Hormone receptor positive breast cancer (HR+) was defined as ER+, PgR+/− and HER2-. HER2 positive cases (HER2+) were defined as ER+/−, PgR+/− and HER2+. TNBC was defined as negative for all above mentioned receptors (ER-, PgR-, HER2-).

Blood samples were retrieved from each patient at BL and after approximately 1, 3, 4, and 6 months of first-line therapy. In patients where treatment was changed to second-line therapy (n=3), blood samples were taken and analyzed at more frequent time-points and for a longer period. A description of the study design is found in Figure 1. All treating physicians and research nurses were blinded to the CTC results. The study was approved by the ethical committee of Lund University (EPN 2010/135) and all patients signed a written informed consent.

The three cell lines MCF7, SKBR3, and JIMT1 spiked into healthy donor blood (HDB) were included as positive controls for cancer gene expression. All cell lines were obtained from the American Type Culture Collection (ATCC/LGC Standards GmbH, Wesel, Germany) and grown as previously described [77] before spiked into HDB. Both positive (cell lines) and negative (only HDB) controls were analyzed in parallel with patient samples using AdnaTest and subsequent gene panel analysis.

### CTC analyses

From each patient, three blood samples were analyzed at every time point specified above. One sample was drawn in a CellSave tube for analysis in the CellSearch system (Janssen Diagnostics). With this system, enrichment and enumeration of CTCs in 7.5 ml whole blood was performed by immunological separation of CTCs from other blood cells [2]. Briefly, antibodies against EpCAM attached to small ferrofluid particles were used to identify CTCs by the epithelial origin of breast cancer tumor cells. By applying a magnetic force, CTCs were enriched in a background of leukocytes and further stained by antibodies against CK 8/18/19, the leukocyte antigen CD45 and with nuclear DAPI staining. CTCs were identified in the CellTracks II system (Janssen Diagnostics) as cells with the phenotype DAPI+/CK+/CD45-. In this study, CellSearch was used to count the number of CTCs in each sample. A CTC count of ≥5 CTCs/7.5 ml whole blood was used as cut-off for positivity.

Two blood samples were drawn in EDTA or AdnaVacutainer (QIAGEN) tubes for characterization of CTCs on a molecular level. After blood draw, the tubes were kept in 4°C until analyses. The AdnaTest EMT1 and AdnaTest EMT2 kits (QIAGEN) were used for enrichment of CTCs from 5 ml whole blood, respectively. Both kits employ immunomagnetic capturing of CTCs but different antigens are used, the EMT1 kit has antibodies against EpCAM and MUC1 whereas the EMT2 kit has antibodies against EpCAM, HER2, and EGFR. It has been suggested that the EMT2 kit can identify more mesenchymal CTCs that have passed through EMT [78]. The enriched CTCs were purified and lysed to retrieve mRNA. mRNA was transcribed into cDNA with AdnaTest and the commercially available molecular assay AdnaTest Breast Cancer was applied to analyze the gene expression of *EpCAM* (*GA733-2*), *MUC1*, *HER2*, and *beta-actin* in a multiplex PCR analysis. The test was considered positive if a tumor associated transcript (EpCAM, MUC1, or HER2) was detected by Agilent 2100 Bioanalyzer (Agilent Technologies, Santa Clara, CA, USA) with peak concentration ≥15 ng/μl using DNA1000 kit.

Patients were classified as “CTC positive” if they were identified as CTC positive with either CellSearch (≥5 CTCs in 7.5 ml blood) or AdnaTest (EpCAM, MUC1, or HER2 ≥15 ng/μl in 2×5 ml blood).

### Gene expression and Heatmap

cDNA from AdnaTest EMT1 and AdnaTest EMT2 were further analyzed for expression of 38 genes associated with breast cancer and nine reference genes. Genes of known importance for proliferation, metastasis, angiogenesis, EMT, stem cells and treatment resistance were chosen. A list of all included genes can be found in Table 3. The gene panel has been developed by TATAA Biocenter and the analysis, including pre-amplification with the PreAmp GrandMaster® mix, was run by TATAA Biocenter. Duplicate samples were run for all markers on a BioMark™ System (Fluidigm) and correction for contamination of genomic DNA was done by the ValidPrime® assay (TATAA Biocenter).

Pre-processing and analysis of qPCR data for all gene panel markers (Table 3) was done in GenEx software (MultiD Analysis, Gothenburg, Sweden). Of the nine reference genes included in the analysis, the two genes *RPLP* and *ACTB* were found to be most stable by the Normfinder algorithm [79] and were used for normalization of all experimental markers. Cq-values reflect the number of amplification cycles necessary to reach detectable gene expression and are thus negatively correlated with the amount of original cDNA. Cq-values higher than 26 were replaced by 26 and qPCR replicates were averaged before normalization by reference genes. Technical replicates of positive and negative controls from different plates as well as technical replicates for each sample were averaged. Missing data were replaced by highest value +4 for each gene to account for non-detectable amounts of original cDNA. Data was transformed into relative quantities and converted into log scale with base 2. For creating heat-maps data were mean-centered along genes.

As described above, two blood samples were taken at each time point and analyzed in parallel with the EMT1 and the EMT2 kit. The highest expression of each marker from the EMT1 or EMT2 kit was used for further statistical analysis since this value was considered to best reflect positive CTC capture. However, EMT1 and EMT2 derived gene expression data were also analyzed separately for individual patients. Heatmaps were created separately for samples taken at BL, for all samples in the cohort and for each patient individually, using GeneEx software (MultiD).

### Statistical analyses

The Mann-Whitney U-test was used to compare the distribution of continuous variables between patients with CTCs and without CTCs. For categorical variables, Pearson Chi-square test or Fisher's exact test was used, the former for cross tables with minimum expected cell count ≥5 and the latter for cross tables with at least one expected cell count <5. To compare the results from CellSearch and AdnaTest the chance-adjusted agreement value Kappa (κ) was calculated. Correlation between CTC number and expression of conventional CTC markers was measured by the Spearman rank correlation coefficient (R_S_).

For statistical analyses, the expression of all markers was dichotomized into present or absent (0 vs 1) due to the large amount of absent Cq-values representing absence of cDNA in the sample. BL gene expression was analyzed for association with breast cancer subtype, stage IV at initial diagnosis, CTC positivity, metastasis localization and number of metastases. Gene expression in FU samples was mainly investigated in spaghetti-plots by graphic evaluation in subsets of patients, or analyzed statistically for association with PD or death (only *HER2* and *ALDH1*). For analyses of expression in FU samples, only patients with samples from at least three time-points were included.

FU time was calculated from BL sample taking date to the last date of clinical FU (for patients alive at the last revision of the patient´s record) or death. The primary end-point was PFS and the secondary end-point OS, both calculated from BL blood sample taking date, but to the date of PD or death, respectively. Early PD was defined as clinical progression within six months from BL sample taking date. The impact of BL gene expression on survival was evaluated using Kaplan-Meier (KM) analysis and the evidence against the null hypothesis of equal survival summarized by *P*-values from log-rank tests. A conventional log-rank test does not perform well when the sample size is small and especially not when the sizes of the groups compared are very different. Hence, a permutation based log-rank test, implemented in the R-package exactRankTests, was used when the size of the smallest group was lower than five. The *P*-values calculated using the permutation method were in general much larger than those from a conventional log-rank test. When analyzing time to early PD, the total FU time was censured at six months.

The statistical analyses were done in IBM SPSS Statistics (version 22, IBM, Armonk, NY, USA) and RStudio (version 0.99.484; https://www.R-project.org/). Crude *P*-values are presented, no adjustment for multiple testing. Consequently, a *P*-value should be interpreted as level of evidence against the null-hypothesis. No *P*-value cut-off for significance was applied in the present study.
